# Activated Charcoal: A Highly Potent Legal Alternative for *Vespa velutina* Nest Destruction

**DOI:** 10.3390/insects17040407

**Published:** 2026-04-09

**Authors:** Andreas W. M. Presuhn, Ulrich R. Ernst

**Affiliations:** 1 Independent Researcher, Am Damm 2, 67122 Altrip, Germany; velutina.presuhn@gmail.com; 2 Independent Researcher, Blumenstr. 5, 73760 Ostfildern, Germany

**Keywords:** invasive species, biodiversity, pest management, allergies, biocide, bee keeping, hornets, activated carbon

## Abstract

The invasive yellow-legged hornet *Vespa velutina nigrithorax* has spread across Europe following its accidental introduction into France in 2004. This species adversely affects biodiversity, apiculture, pomiculture and viticulture, and human health. Current management relies predominantly on nest destruction; however, manual removal is often logistically challenging and costly because nests are typically located high in trees (up to 30 m), frequently necessitating vehicle-mounted lifts. Ground-based application of biocides using long injection lances is comparatively rapid and inexpensive, but in many countries, insecticides are not permitted because the products are not specifically authorized for hornet control. Consequently, alternative approaches are needed. Here, we evaluated the efficacy of activated charcoal for nest destruction in *V. v. nigrithorax*. We injected 145 nests with 50–100 g of activated charcoal and subsequently destroyed the nests. One week later, we assessed worker survival and the establishment of new nests. Emergency nest construction by surviving workers was observed in three of 145 cases (2.1%). This rate was comparable to that observed following insecticide treatment (two of 136 cases; 1.5%). Activated charcoal therefore appears to be similarly effective to insecticide-based control while offering advantages in terms of environmental compatibility, user safety, ease of handling, and legal applicability in Europe. Activated charcoal may represent a practical alternative to manual nest removal and unauthorized insecticide use.

## Simple Summary

The rapid expansion of the invasive yellow-legged hornet (*Vespa velutina nigrithorax*) across Europe presents a critical challenge to biodiversity, apiculture, and public health. Current management strategies are often constrained by the lack of authorized biocides for hornet control and the logistical complexity of manual nest removal. This study evaluates the efficacy of activated charcoal as a highly potent, biocide-free alternative for nest destruction. In a cross-border field study conducted in Germany and Switzerland, 145 secondary nests were treated with 50–100 g of activated charcoal dust using pneumatic lance systems, followed by mechanical brood removal. The protocol achieved a 98% nest inactivation rate within seven days (142/145 nests), demonstrating efficacy statistically comparable to a standard cypermethrin-based insecticide treatment (98.5%). Microscopic analysis of treated specimens confirmed a physical mode of action, revealing severe occlusion of spiracles and tracheal blockage that leads to rapid functional impairment and respiratory failure. Unlike synthetic insecticides, activated charcoal leaves no toxic residues and faces fewer regulatory hurdles. These findings establish activated charcoal as a scalable, legally robust, and environmentally compatible tool for the area-wide management of *V. velutina*.

## 1. Introduction

Invasive species are recognized by IPBES (Intergovernmental Science-Policy Platform on Biodiversity and Ecosystem Services) as a major driver of biodiversity loss [1]. The yellow-legged hornet (*Vespa velutina nigrithorax*) is among the most rapidly expanding invasive hymenopterans of the past two decades and poses a particular challenge due to its high spread rate and impacts on biodiversity, human health, and the economy [2]. Following its introduction to South Korea (2003) [3,4] and the first record in Southern Europe/France (2004), the species spread rapidly across Europe [5]. By 2025, occurrences have been documented in Asia, Europe, and North America [6]; additional records have been reported for North Africa (Algiers, Algeria) [7,8] and for Auckland, NZ [9]. Thus, the species has been recorded on four continents and in New Zealand.

*V. velutina* rapidly establishes large secondary nests and is notorious for its intense predation on managed honey bees (*Apis mellifera*); however, this opportunistic generalist predator [10] also heavily preys on most other arthropods, including diptera, other social wasps, and bumble bees (*Bombus* spp.) [10,11,12,13,14,15]. Consequently, the unchecked proliferation of this invasive hornet threatens not only commercial apiculture, but also critical ecological functions such as wild plant pollination [16,17] and natural population control of native entomofauna.

In Spain, approximately 5.1–20.5% of apiary revenue is spent on controlling *V. velutina* [18]. Under a scenario of maximal spread in France, annual losses of up to €30.8 million due to colony mortality alone have been projected [19]. Reported impacts also include damage to viticulture [20] and insufficient pollination associated with hornet presence [17]. For Germany, annual management costs under a maximal spread scenario have been modelled at ~€5 million, compared with ~€11.9 million in France [21], where projected beekeeping-related losses are substantially higher [22]. These estimates do not include potential damage to agriculture (wine and fruit production) or public health and broader economic costs associated with stings [23]. Moreover, ecological impacts are likely considerable [10,11,12,13,14,15], although monetizing biodiversity remains challenging [10]. In Europe, management accounts for only about 6% of total costs attributed to invasive organisms, implying that prevented damage can greatly exceed control expenditure [24].

In Germany and parts of the European Union (EU), operational control is constrained by conservation and biocidal-product regulations. Trapping systems, including electric “harps” [25,26,27,28], are legally problematic because the capture of protected non-target species (e.g., *Vespa crabro*)—even as bycatch—is not permitted under nature conservation law. Given the repeatedly documented lack of selectivity of passive traps [29,30,31,32] and their limited contribution to sustained population suppression [33], their use is generally not recommended. Defensive measures at apiaries (e.g., entrance guards and nets [2,34,35]) reduce colony risk but do not constitute population control [19]. Relocating colonies to hornet-free areas is often impractical in regions with widespread establishment and can impose additional costs that should be minimized under EU Regulation No. 1143/2014 of the European Parliament and of the Council of 22 October 2014 on the prevention and management of the introduction and spread of invasive alien species [36]. Preventing spread to other Member States of the EU likewise requires management approaches that include legally viable nest elimination [36]. Toxic bait helped reduce the number of *V. velutina nigrithorax* at beehives [37]; however, its use is likely in conflict with EU regulations and poses environmental risks. The same applies to candidate insecticides [38,39]. To date, systematic nest detection and removal remains the only consistently effective strategy [2]. While management in Europe is largely focused on containment and impact mitigation due to the widespread establishment of the species [39], regions facing recent incursions employ aggressive eradication protocols. On islands, the chances for eradication are higher than on the mainland where invasions from neighbouring regions will inadvertently occur [40].

In the United States, following the 2023 detection of *V. velutina* in Georgia [6,41], authorities implemented ‘trap-treat-release’ methodologies [42,43] (similar to the ‘Trojan horse method’ [44]), utilizing topical applications of fipronil to achieve targeted colony collapse, alongside physical nest eradication [45]. Similarly, in New Zealand, the Ministry for Primary Industries relies on intensive trap grids, targeted ground searches, and insecticide bait (fipronil [46], cf. [37,45]), combined with electronic tracking telemetry (the ‘Judas technique’ [47,48]) to locate and physically destroy nests hidden in dense canopies before establishment can occur [49].

In Japan [50], nests are sprayed with pyrethroids and then manually removed [51] (*fide* [52]); additional insecticides are also under investigation [52]. In Korea, nest destruction is done using fire or poison [3] and unmanned aerial vehicles (UAVs) are being experimentally used to detect nests [53] and spray pyrethrum-based insecticides into high-altitude nests [54].

Regardless of the geographic region, the regulatory environment, or the phase of invasion, the localization and subsequent destruction or chemical inactivation of the nest remains the universal endpoint of effective management strategies.

Conversely, in the hornet’s native range across Southeast Asia (reviewed in [3]), populations are regulated by natural ecosystem dynamics (e.g., competition with other hornet species [50,55,56,57,58]) and these hornets have not caused substantial economic problems [57]. In fact, *V. velutina* is even reared for food production [59] and control is usually limited to defensive measures [60]. Practices include localized apiary trapping [61] and manual defensive swatting [62].

A cost- and time-efficient method is the injection of insecticides using lance systems. However, in Germany and several other EU countries, there are no broadly authorized products for general use against *V. velutina* [39]; available biocides are often restricted to use “in, on, or around” buildings. In addition, biocide use raises concerns about environmental residues and secondary poisoning of non-target organisms [63], especially when nests are not removed [39,64]. Similar concerns apply to “Trojan horse” baiting approaches, in which a biocide is applied to a worker in the expectation that it will be transported into the nest; environmental exposure, the fate of treated individuals, and secondary poisoning risks are difficult to quantify, particularly when persistent and highly toxic substances (e.g., neonicotinoids) are involved. Purely mechanical approaches such as manual nest removal with vehicle-mounted lifts are limited by high operational costs, equipment logistics, terrain constraints, safety requirements, specialized personnel, and seasonal availability; accident risk is non-negligible. Shooting nests [34] is ineffective because nests are often repaired and reproductive interruption is not assured, and scattered workers may establish emergency nests that can increase public health risks. CO_2_-based immobilization is considered an unauthorized biocidal application under German biocidal-product provisions because biocides are legally defined as agents that chemically interact with the target organism [65]; CO_2_ effects are mediated by reversible disruption of pH-dependent metabolic processes [66]. Steam treatment [67] does not require approval as a biocide because of its physical mode of action; however, it imposes substantial technical and occupational-safety demands [68,69]. Overall, high equipment and maintenance costs, training requirements, and safe application constraints can be prohibitive for large-scale control under limited budgets.

For *V. velutina* nest control in public settings, no biocidal products are specifically authorized within the EU [38,39,70]; transitional and case-by-case arrangements are heterogeneous and often impractical for area-wide programmes [71]. Diatomaceous earth has a physical mode of action but is classified as a biocide [72], shows weather-dependent performance [73] and is currently not authorized for invasive hornet control in Germany and Switzerland. In contrast, air-assisted lance systems provide a logistically efficient means of applying powdered agents with predictable one-time acquisition costs and high operational flexibility. In Rhineland-Palatinate (Germany), control of secondary nests in 2024 relied on such systems because they enabled flexible, cost-efficient operations with a reported throughput of up to 15 nests per person per day, depending on nest location. Given that many nests occur in public areas, an applied substance must be legally unproblematic and either meet biocidal-product requirements or act outside the scope of biocidal regulation.

Accordingly, a biocide-free, legally robust, scalable, and effective method is required. Usability and, if possible, animal-welfare compatibility are additional advantages, as they increase societal acceptance. Activated charcoal meets these criteria: it is chemically inert and acts via mechanical–physical processes. Physical dusts with low unit costs can support continued control of *V. velutina*, consistent with the obligation to continue management under Regulation (EU) No. 1143/2014 [36]. The proposed mechanism (adsorption/surface effects) involves disruption of the cuticular lipid barrier, impairing water balance and leading to mortality [74]. The rapid functional impairment observed is also compatible with a suffocation component through mechanical occlusion of spiracles [75]. In laboratory and applied studies, activated charcoal showed effects comparable to or exceeding those of diatomaceous earth against the pharaoh ant *Monomorium pharaonis* [74]. Because its action is mechanical, no biocidal authorization is required (subject to national competent-authority classification) [76]. Activated charcoal leaves no toxicological residues and is generally considered environmentally benign. Activated charcoal is a chemically inert material structurally analogous to natural black carbon, and is characterized by high environmental persistence without inherent toxicity [71,77,78,79,80]. It is allowed to be used as a food additive, as a feed additive, in cosmetics, for medical use and for water purification, indicating its low oral and contact toxicity (reviewed in [81]). Its presence in soil reduces harmful nutrient leaching and contributes to the stable organic carbon pool [82,83]. Ecotoxicological assessments [80] have demonstrated that activated charcoal particles in non-polluted environments do not negatively affect the survival or growth of key organisms and are helpful in contaminated environments [80]. Furthermore, the high adsorption capacity of activated charcoal can facilitate the sequestration of existing pollutants, thereby reducing their overall bioavailability. Given its parallels with biochar used as a soil conditioner [84,85], the incidental release of activated charcoal dust during pest control operations is expected to have a negligible ecological impact. Activated charcoal is neither toxic nor harmful: the LD50 for oral uptake is 10 g/kg, and there is no evidence of harm to water organisms [77,78,86,87]. Consequently, the application of activated charcoal in this context poses no significant risk to local ecosystems or soil health [88,89]. However, very fine dust can affect human health if inhaled chronically [90]. Therefore, appropriate personal protection equipment, consisting of particle filtering mask (FFP2) and gloves are required during the handling of activated charcoal. As a fundamental matter of responsible self-personal protection when working with various substances and preparations—especially insecticides—the user should always read the Safety Data Sheet (SDS) of the specific product being used and implement the recommended protective measures [91]. The aim of this study is to describe activated-charcoal nest control under field conditions and to evaluate its effectiveness using a standardized application protocol (including brood removal). We show that, when applied correctly, activated charcoal achieves an effectiveness close to that of biocides.

## 2. Materials and Methods

### 2.1. Prospective Field Efficacy Study: Setting and Case Selection

A prospective field study was conducted during routine control operations in Rhineland-Palatinate, Germany, in 2024. Fifteen secondary nests of *Vespa velutina* were included if they were (i) reachable with an injection lance, (ii) readily observable, and (iii) associated with low immediate risk to unprotected bystanders.

For external confirmation, an independent dataset was collected in Switzerland using the same protocol (*n* = 25). Nest height was estimated from the number/length of extended lance segments up to the nest edge. Nest volume was estimated visually using prior calibration with rotation ellipsoids derived from reference nests. Nests where the surrounding area could not be adequately cordoned off were not included. No experiments were conducted during rain.

The activated charcoal used was a black powder (S3 Handel und Dienstleistungen UG, Bad Oeynhausen, Germany) with a particle size < 40 µm, BET (Brunauer–Emmett–Teller) surface area of approximately 900 m^2^/g, pH of 9–12, water content ≤ 10%, ash content ≤ 9% (CAS 7440-44-0; EC 231-153-3), and estimated cost of ~€30 per kg. Field applications were performed with a “Buzz Busters” lance system (Buzzbusters, Guillerval, France, Supplementary Figure S1), which provides a basic reach of 20 m that is extendable to ~30 m; it is operated with a battery-powered compressor (Einhell TE-AC 36/6/8 Li, Landau an der Isar, Germany) and equipped with a moisture separator and emission-reducing application tips. Personal protective equipment consisted of a particle half mask (FFP2 (filtering facepiece 2)), gloves, and safety goggles during filling of the pressure reservoir, and a full hornet protective suit, gloves, and face protection/veil, along with cordoning equipment, during injection into a nest.

Field treatments followed a standardized application protocol. Activated charcoal was preferentially applied via the nest entrance; when the entrance was inaccessible, the nest envelope was punctured and the dust was introduced from below. Working pressure was adjusted to approximately 1 bar per 10 m of working height and increased stepwise as needed. Each nest received 50–100 g of activated charcoal delivered as an initial application followed by a second application 15 min later. The brood was removed by mechanically extracting combs using the “destroyer” attachment of the lance system (Supplementary Figure S1); removed comb material was collected and disposed of. Where necessary, remaining envelope fragments were re-dusted to target adults still present. Operations were not conducted during rain, and deployments under strong wind were avoided.

Field outcomes were defined a priori: The primary outcomes were (i) acute functional impairment, recorded as flight incapacity and/or falling to the ground within the first hour after application, and (ii) nest inactivation, defined as the presence of no more than 1–2 adults during a 10 min observation period on day 7 and the absence of flight activity by day 10. Secondary outcomes included whether the nest was repaired or continued operation (yes/no), the formation of emergency nests in the vicinity, and observable brood changes (e.g., discoloration or dust deposition). We recorded the applied dose (g), working time (min; from the beginning of setup to completion of teardown), and working height (m).

#### 2.1.1. Data Collection and Quality Assurance

Observations were recorded using a standardized schedule with time stamps (0–60 min, day 1, day 7/10), with two observers whenever feasible. Fallen adults were not collected to avoid interfering with the course of effects. Equipment function (pressure build-up and nozzle fit) was checked before each deployment.

#### 2.1.2. Preliminary Study on the Need for Mechanical Destruction

In order to test whether the application of activated charcoal alone without subsequent mechanical destruction of the nests would be sufficient, we left three nests undamaged after the application. We stopped this trial because in all three cases, the nests were still active 10 days after the treatment.

### 2.2. Independent Field Validation (Switzerland, 2025) and Insecticide Comparator

For external validation, two independent experimenters treated 108 secondary nests in the cantons of Basel-Stadt and Basel-Landschaft, Switzerland, in 2025 using the identical protocol (Supplementary Figure S2). The outcomes were documented in a standardized format; success control was performed on day 7 only (criterion: nest inactivation).

For contextual comparison, the outcomes were contrasted with those from 136 nests treated in 2024 in Rhineland-Palatinate, Germany, with a biocide (Vespa; 0.5% cypermethrin; Armosa Tech SA, Engis, Belgium) using an analogous operational approach (Supplementary Figure S3).

### 2.3. Sample Preparation for Microscopy (Mechanistic Assessment)

#### 2.3.1. Spiracle Preparations

We collected hornets from several nests from the ground 24 h after nests had been treated with activated charcoal. To assess morphological changes, thoraces were separated and embedded in UV-curable resin (MOCOBO, UV Resin, Hard Type, Sea&Mew Consulting GmbH, Neumarkt, Germany) to improve stability and handling. Resin blocks were mechanically ground (Parkside, PTSG 140 C2, Neckarsulm, Germany) until spiracles and adjacent tissue remnants were exposed. Samples were incubated for 5 days in 1 M NaOH (S3 Chemicals) to macerate the organic material and dissolve the resin. After soft tissue dissolution, the remaining chitinous thoracic structures were rinsed twice with demineralized water.

The specimens were bleached in 5% H_2_O_2_ (S3 Chemicals) for 30 min in a water bath at 80 °C and then left in solution for an additional 48 h until sufficient transparency for transmitted-light microscopy was achieved. As activated charcoal is chemically inert to H_2_O_2_, it remained visible as a distinct black material within tissues. Final mounting was performed in Kaiser’s glycerol gelatine (S3 Chemicals).

#### 2.3.2. Tracheal Preparations

To minimize contamination during dissection, hornets were repeatedly washed in 0.1% SCS (sodium coco sulphate surfactant solution; Salandis eGBR, Greifswald, Germany) until no external charcoal residues were visible. Thoraces were then sagittally bisected. Primary tracheae and air sac membranes were dissected under water and mounted in Kaiser’s glycerol gelatine (S3 Chemicals).

## 3. Results

### 3.1. Prospective Field Series (Germany 2024; n = 15 Secondary Nests)

**Figure 1:**
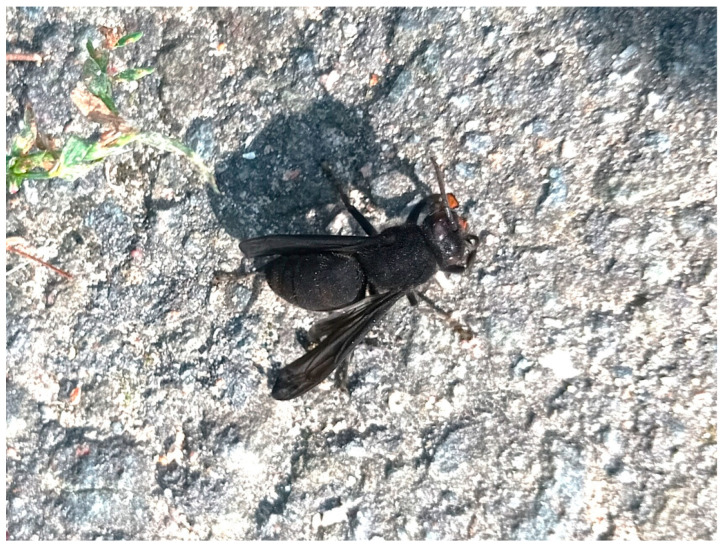
Individual *Vespa velutina nigrithorax* worker covered in activated charcoal. After application of activated charcoal, workers (and where present, gynes and drones) leave the nest and fly or fall to the ground, where they often become motionless within minutes.

During the acute observation period (0–60 min), many workers exited the nest immediately after application; within minutes, most individuals showed marked flight impairment and fell to the ground (Figure 1) (cf. Supplementary Material S4). Overall nest traffic declined sharply and was strongly reduced within approximately 1 h. By day 1, numerous dead individuals had accumulated beneath treated nests. At follow-up, at three of 15 nests, residual activity was observed on day 7 (~1–2 adults per 10 min), but by day 10, no flight activity was detected. Across all cases, no nest repair or continued operation was observed (15/15).

Operationally, 50–100 g of activated charcoal was applied per nest (mean 71 g). The total on-site time ranged from 40 to 70 min (mean: 54 min, including setup and teardown). The mean working height was 9 m and the mean estimated nest volume was 46 L, although treatments at heights of 20–30 m were feasible. No stings were recorded during operations, and no deployments were conducted during rain.

In all three cases where we purposefully did not mechanically destroy the nest after treatment with activated charcoal, activity at the nest persisted 10 days after treatment.

### 3.2. Cross-Site Operational Parameters (Germany 2024 vs. Switzerland 2025; Subset Comparison)

A subset comparison (Germany: *n* = 15; Switzerland: *n* = 25) indicated that Swiss deployments targeted higher nests on average (18 ± 8.2 m vs. 9.2 ± 3.8 m), whereas estimated nest volume was larger in the German sample (46 ± 5.8 L vs. 38 ± 7.8 L). Despite smaller nests, the mean charcoal mass and working time were higher in Switzerland (96 ± 14 g; 86 ± 48 min) than in Germany (71 ± 12 g; 54 ± 9.1 min). The mean volume-specific dose was 2.6 ± 0.4 g/L in Switzerland vs. 1.6 ± 0.3 g/L in Germany (+66%) (Table 1 and Table S1).

Within the observed autumnal range of temperature and relative humidity reported for both datasets, no efficacy differences were apparent in this subset (all nests were inactive on day 7; no repairs or emergency nests were observed). Because no failures occurred in this subset and environmental variability was limited, no weather- or dose-dependent thresholds could be inferred.

### 3.3. Field Efficacy of Activated Charcoal and Comparison with a Biocide Dust (Day 7 Endpoint)

After describing cross-site differences in deployment conditions and dosing intensity (Section 3.2), we assessed efficacy based on the day 7 terminal endpoint (nest inactivation) across the pooled protocol-adherent activated-charcoal dataset (Germany and Switzerland) and compared outcomes with an operational cypermethrin-based dust series. Across all protocol-adherent activated-charcoal treatments (total *n* = 145 secondary nests), 142/145 nests reached the terminal endpoint on day 7 (97.9%), whereas 3/145 nests remained active and/or showed repair (2.1%) (Table 2). The pooled dataset comprised the German prospective series (2024; *n* = 15; all inactive at day 7), a Swiss confirmation series (2025; *n* = 25; all inactive at day 7; operational-parameter data available for a subset reported in Section 3.3), and an independent Swiss field test (2025; *n* = 105). In the independent Swiss field test, 102/105 nests were inactive on day 7 (97.1%), while 3/105 showed persistent activity and/or repair (2.9%); these non-terminal outcomes were characterized by small repair structures despite otherwise protocol-adherent treatment. In a separate ancillary test evaluating whether brood removal could be omitted, three additional nests were treated with activated charcoal without brood removal (protocol deviation); all three remained active and exhibited repair on day 7 (0/3 inactivated), supporting brood removal as a required protocol element. For contextual comparison, a cypermethrin-based biocide dust applied via the same lance system in Germany (2024; *n* = 136) resulted in 134/136 (= 98.5%) nests being inactive on day 7, whereas 2/136 (= 1.5%) nests showed repair and/or emergency nest formation. This result (two live nests out of 136) is not significantly different from the results of the activated charcoal trial (three live nests out of 145) (Fisher’s exact test, *p* = 1.000). Operationally, cessation of nest traffic occurred earlier than with activated charcoal, consistent with the expected rapid knockdown effect of the active ingredient (Table 2).

### 3.4. Microscopy

**Figure 2:**
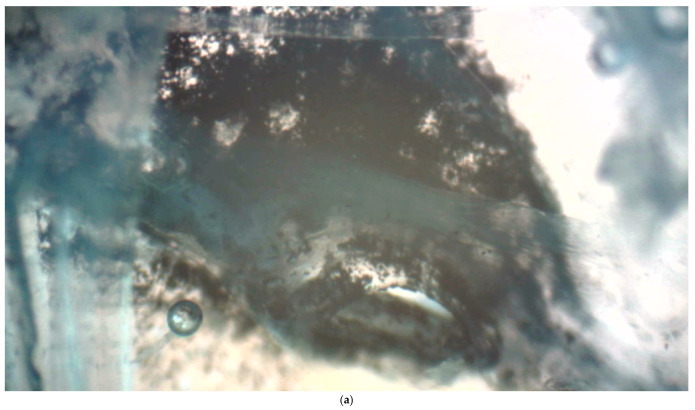

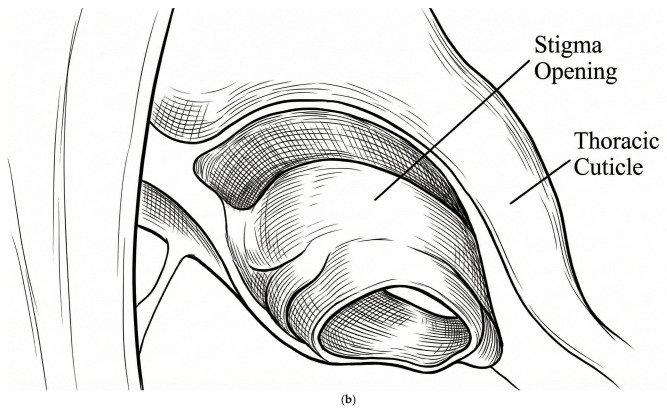
(**a**) Dense deposit of activated charcoal particles outside of a spiracle in the thorax. Seen from within the body, charcoal particles accumulate around the spiracle; (**b**) schematic drawing clarifying the location of the spiracle.

**Figure 3:**
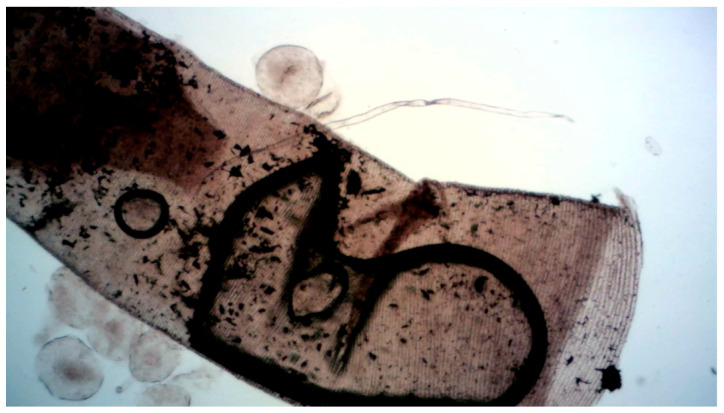
Particles of activated charcoal entered the tracheal system. Shown is the main trachea in the thorax. Circular structures are artefacts (air bubbles). After treatment with activated charcoal, individuals died and were kept frozen until dissection. This trachea was taken from the thorax of a hornet that died after the treatment protocol. The thorax was dissected under water, and the trachea was detached from the stigma’s atrium using forceps and transferred into glycerol for light-microscopic examination.

Transmitted-light microscopy of macerated and bleached thoracic sections revealed dense accumulations of activated charcoal particles within the spiracular atrium (Figure 2). Deposits persisted despite repeated washing and H_2_O_2_ treatment, and the atrial region appeared mechanically compromised by particle loading. We detected activated charcoal throughout the tracheal system, including partial to extensive occlusion of the main tracheae (Figure 3). Occasional particle adhesion was also observed on air sac membranes.

## 4. Discussion

### 4.1. Principal Findings

Under a standardized application protocol, activated charcoal rapidly incapacitated adult *Vespa velutina*, resulting in the total cessation of nest activity within 7 days.

### 4.2. Mode of Action, Welfare Considerations, and Safety

Across protocol-adherent field deployments, nests were reliably inactivated without behavioural patterns commonly interpreted as pronounced nocifensive responses, such as marked abdominal curling, vigorous writhing, or abrupt aggression peaks. While the absence of these signs does not definitively prove the absence of pain or suffering [68,69], the evaluation of invasive species management should prioritize animal welfare and operator safety alongside non-target toxicity and environmental persistence [38,39]. This is particularly relevant given that several conventional insecticides pose significant health risks to mammals and humans (reviewed in [39]).

The observed sequence of disorientation, loss of flight capability, immobilization, and death supports a mechanical–physical mode of action. Two complementary mechanisms [92] are consistent with our data: (i) disruption of the cuticular lipid barrier, leading to impaired water balance (reviewed in [74]), and (ii) (partial) occlusion of spiracles and airways, resulting in respiratory compromise. Efficacy likely increases during phases of high worker activity; nest disturbance induces elevated ventilation associated with flight, which may enhance particle intake from dust-saturated air and accelerates functional impairment.

From an operational perspective, health hazards for the operator are minimized through appropriate personal protective equipment (gloves, FFP2 mask, and goggles) and safety measures such as cordoning and weather-adapted deployments. Nevertheless, all users should strictly adhere to the Safety Data Sheets (SDSs) and implement recommended protective measures [91].

Effective secondary-nest control also requires consideration of colony relocation dynamics. If migration from the primary to the secondary nest is ongoing during treatment, returning individuals may repopulate the site, potentially leading to repairs or the construction of small emergency nests. Following queen loss, such emergency nests may become male-biased if workers begin laying unfertilized eggs.

### 4.3. Mechanistic Evidence from Microscopy

Microscopic analysis confirmed the uptake of activated charcoal into the respiratory system. Persistent particle loads in the spiracular atria indicate mechanical impairment at the entry of the tracheal system, while charcoal within the main tracheae—including partial to extensive luminal occlusion—supports impaired gas exchange. Particle adhesion on air sac membranes further aligns with respiratory insufficiency as a relevant driver of lethality. These findings match the observed phenotype of rapid loss of flight performance followed by immobilization (cf. Supplementary Material S4). Tracheal obstruction [93] may represent a dominant pathway under the high airborne dust conditions generated during nest treatment [43].

While the inhalation of plant-derived insecticides such as derris (active ingredient: rotenone) plays a role in arthropod toxicity, its mechanism is not based on physical obstruction [94]. In contrast to our finding, earlier studies on the much smaller bean weevil (*Acanthoscelides obtectus*, 2–5 mm) found no evidence of respiratory impairment [95]. Similarly, studies using sodium fluoride (NaF) or magnesium carbonate (MgCO_3_) excluded tracheal blockage or failed to detect particles within the tracheae [75,96,97]. Possibly, insects in flight—which have significantly higher ventilation rates—are more affected than crawling insects.

Cuticular disruption also likely contributes to mortality, particularly in individuals with lower respiratory uptake. The relative importance of airway occlusion versus desiccation likely depends on the balance between internal blockage and whole-body coverage.

Furthermore, sensory impairment may contribute to early disorientation. Activated charcoal can coat compound eyes and antennal sensilla, potentially reducing visual and olfactory function. Dust-related interference with antennal olfaction has been demonstrated in flies [98] and may be particularly consequential in eusocial insects that rely on chemical communication for colony cohesion and coordinated defence.

### 4.4. Operational Feasibility and Cost Considerations

The approach is material-sparing and operationally efficient: the treatments required only 50–100 g of activated charcoal per nest with mean on-site times of 54 min (Germany) to 86 min (Switzerland). Lance-based application to heights of 20–30 m reduces dependence on vehicle-mounted lifting equipment. The protocol is readily implemented as a standard operation procedure (SOP)—including pressure scaling (~1 bar per 10 m working height), follow-up applications after 15 min, and brood removal—facilitating training, quality assurance, and scaling. Furthermore, activated charcoal represents a highly cost-effective alternative to specialized biocides as it is an inexpensive material available in bulk quantities globally.

### 4.5. Contextualization Relative to Existing Approaches

Compared with synthetic insecticides, activated charcoal does not produce immediate knockdown but instead induces rapid functional impairment and a predictable colony collapse within a defined post-treatment window.

Relative to diatomaceous earth, the activated-charcoal approach is intended as a biocide-free strategy in jurisdictions where mechanically acting dusts are not classified as biocidal products; it may also be less sensitive to weather-related limitations than some other dust-based interventions. Laboratory and applied studies in other insect systems have reported comparable or superior performance of activated charcoal relative to diatomaceous earth [74,75,99,100].

A practical advantage is the absence of toxicological residues, which minimizes concerns about secondary poisoning of non-target organisms [63]. Where appropriate, remaining treated nest material may therefore be left in situ and remain available to native scavengers. However, mechanical removal of brood combs was crucial to the success of the method. In contrast, insecticide-treated nests are usually not removed, trading working speed for higher environmental risks [39].

Finally, resistance evolution is expected to be less likely than with neurotoxic insecticides as adaptation would require more complex morphological or behavioural changes [101] than target-site or metabolic resistance mechanisms [102,103,104,105].

### 4.6. Implications and Future Directions

Activated charcoal represents a legally robust, biocide-free, and scalable option for *V. velutina* nest management, particularly where authorized insecticides are unavailable or undesirable. Priority next steps include:

- Controlled comparative trials against alternative mineral/physical dusts (e.g., kaolin and zeolites) and, where legally permissible, benchmark insecticides, using standardized time-to-event endpoints.
- Optimization of dose and particle properties (size distribution, surface area, and deposition).
- Stratified analyses across seasons and colony phases (nest stage, colony size, and migration status).
- Targeted monitoring of non-target impacts and environmental pathways under operational conditions.

## Figures and Tables

**Table 1:** Operational parameters for activated-charcoal treatment (subset comparison).

Parameter (Mean ± SD)	Germany 2024 (*n* = 15)	Switzerland 2025 (*n* = 25)
Nest height (m)	9.2 ± 3.8	18 ± 8.2
Estimated nest volume (L)	46 ± 5.8	38 ± 7.8
Activated charcoal (g)	71 ± 12	96 ± 14
Dose per volume (g/L)	1.6 ± 0.3	2.6 ± 0.4
Working time (min)	54 ± 9.1	86 ± 48

**Table 2:** Day 7 terminal endpoint (nest inactivation) after treatment with activated charcoal (by dataset; pooled protocol-adherent performance; and ancillary brood-removal omission test) and a cypermethrin-based biocide dust comparator.

Treatment/Group	Location (Year)	Protocol Status	*n*	Inactive on Day 7, *n* (%)	Active/Repair on Day 7, *n* (%)	Notes on Non-Terminal Outcomes
Activated charcoal (prospective series)	Germany (2024)	Protocol-adherent (incl. brood removal)	15	15 (100%)	0 (0.0%)	—
Activated charcoal (confirmation series)	Switzerland (2024/2025)	Protocol-adherent (incl. brood removal)	25	25 (100%)	0 (0.0%)	—
Activated charcoal (independent field test)	Switzerland (2025)	Protocol-adherent (incl. brood removal)	105	102 (97.1%)	3 (2.9%)	Small repair structures
Activated charcoal (pooled)	Germany + Switzerland (2024–2025)	Pooled protocol-adherent datasets	145	142 (97.9%)	3 (2.1%)	Repair and/or persistent activity
Activated charcoal (ancillary test)	Switzerland (2025)	Brood removal omitted (protocol deviation)	3	0 (0.0%)	3 (100%)	Flight activity and repair
Biocide dust (0.5% cypermethrin)	Germany (2024)	Operational comparator	136	134 (98.5%)	2 (1.5%)	Repair and/or emergency nest formation

Notes: The day 7 endpoint was recorded as a binary outcome (inactive vs. active/repair). In the German prospective series, nest inactivity was additionally corroborated by absence of flight activity on day 10.

## Data Availability

The original contributions presented in this study are included in the article/Supplementary Material. Further inquiries can be directed to the corresponding author.

## Supplementary Materials

The following supporting information can be downloaded at: https://www.mdpi.com/article/10.3390/insects17040407/s1, Figure S1: Materials used for the injections and physical nest destruction, including extendable lance; Figure S2: Location of nests in Switzerland treated with activated charcoal; Figure S3: Location of nests in Germany treated with biocide; Figure S4a. The brood combs of the nest are upside down, after the adult hornets have been vacuumed off and the nest envelope has been removed; Figure S4b: Dusting with activated charcoal induces rapid loss of flight within minutes (25% within 5 min, 50% within 10 min, 100% in 25 min; blue line) and leads to 99% mortality within 18 h (red dashed line). The hornets were dusted 3 times with activated charcoal within 5 min and then again after 20 min (dashed vertical line), corresponding to the treatment of a nest. Note that the x-axis is logarithmic; Table S1: Data on nest height, nest volume, mass of charcoal used, duration, temperature, and relative humidity; Table S2: Comparison of efficacy of treatment with activated charcoal and biocide. Reference [106] has been cited in Supplementary file.

## Abbreviations

The following abbreviations are used in this manuscript:

EU	European Union
SD	Standard Deviation
