# Supplementary material for: Activated Charcoal: A Highly Potent Legal Alternative for Vespa velutina Nest Destruction
Source: Insects. 2026 Apr 9;17(4):407. doi: 10.3390/insects17040407 (PMC13115649; doi:10.3390/insects17040407)
Supplement: Supplementary file 1 [file insects-17-00407-s001.zip › Supplemental_Material_S4.pdf]

Materials and Methods

Adult mortality assay (flight cage)

We collected a *Vespa velutina nigrithorax* nest on 18.09. 2025. The *Vespa velutina nigrithorax* nest was located in an empty "Mini Plus" type hive. Inside, the nest extended across two hive boxes and contained 5 combs. The high cappings of various cells indicated that the nest had begun producing queens, which also corresponded to the season. The individuals (~400 adults, including a laying queen, young gynes (mesoscutum width >4.5 mm [1]), drones (lack of stinger), and workers) were vacuumed at the entrance and transferred to a flight cage (38 × 38 × 60 cm^3; four mesh sides, one PVC observation window, and a textile floor; QIODAZOO). In a further step, the nest was dismantled (**Supplemental Figure S4a**) to vacuum the remaining individuals and unite them with the swarm in the flight cage.

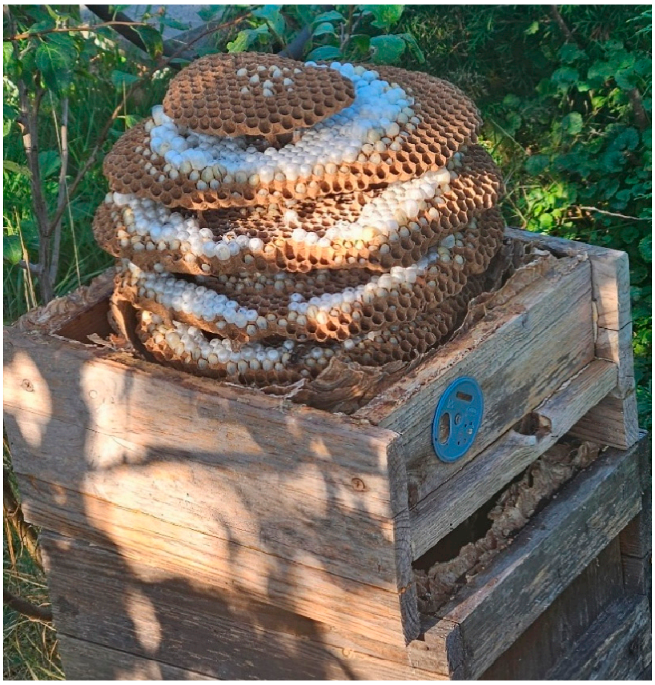

Supplemental Figure S4a. The brood combs of the nest are upside down, after the adult hornets have been vacuumed off and the nest envelope has been removed.

Individuals were allowed to acclimate for approximately 30 minutes. Activated charcoal dust was then applied to the entire cage as “swarm dusting” using a Birchmeier DR5 powder sprayer (Birchmeier Sprühtechnik AG, Stetten, Switzerland). Three bursts were delivered within the first 5 minutes, followed by one additional burst at t = 20 minutes; burst duration was standardized to 2 seconds. Trials were conducted at 23 °C and 65 % relative humidity. Primary endpoints were (i) sustained loss of flight capability (≥30 s) and (ii) mortality. Secondary observations included disorientation, loss of perching ability, impaired motor coordination, grooming behaviour, and rapid immobilization

lacking prolonged neuro-excitatory spasms. We could not quantify the exact numbers of hornets affected at each time point, therefore the proportions are the best available estimation.

Results

Adult mortality assay (flight cage; ~400 adults)

Activated charcoal dusting caused rapid functional impairment followed by near-complete mortality (**Supplemental Figure S4b**). After the second to third dusting (t = 5 min), disorientation and flight disturbance (falls) were evident; at t = 5 min, ~25% of individuals were immobile or showed intensive grooming, and by t = 10 min ~50% were on the cage floor. After the additional application at t = 20 min, all individuals were flightless by t = 25 min. By t = 30 min, ~95% were on the floor with markedly reduced activity; between t = 40 and 60 min most individuals were moribund. At t = 18 h, mortality exceeded 99%, with only occasional very slow leg movements in isolated individuals. No conspicuous prolonged neuro-excitatory spasms nor nocifensive behaviours (e.g., body curling or sudden aggression peaks) were observed; behaviour was dominated by grooming and crawling until immobilization.

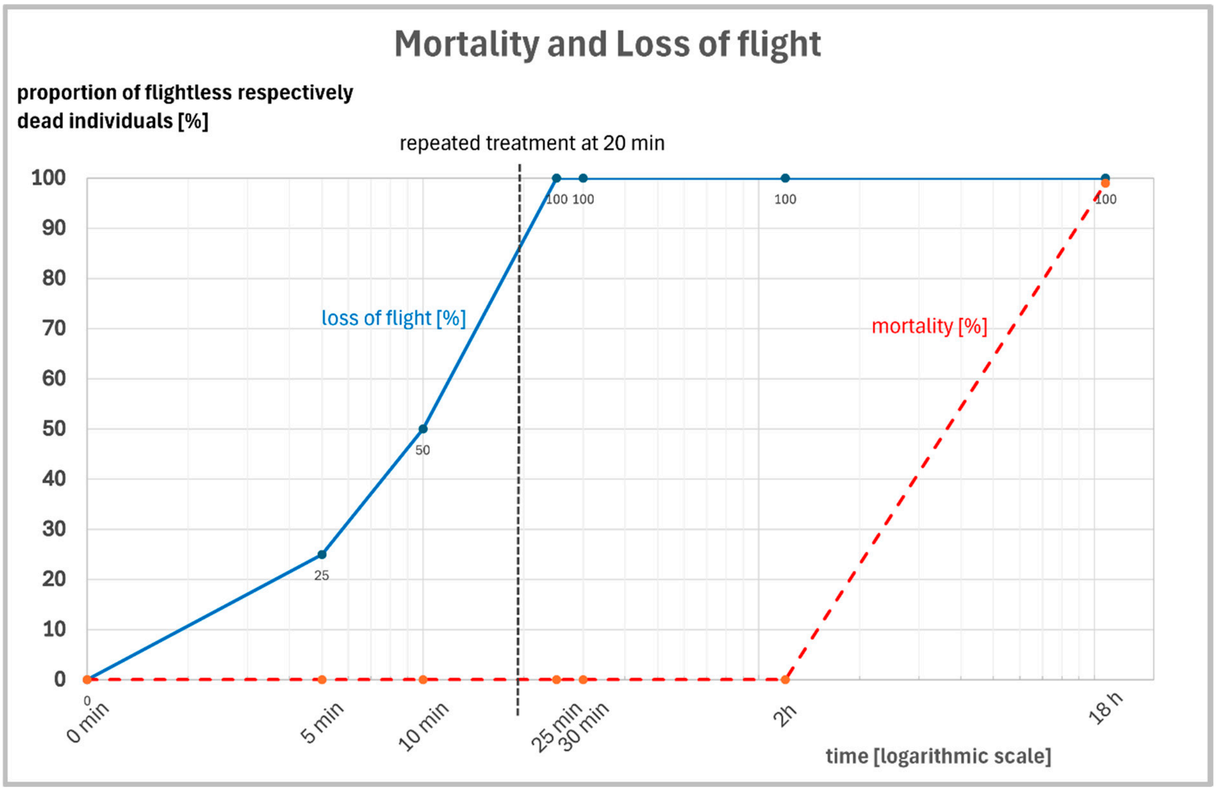

Supplemental Figure S4b: Dusting with activated charcoal induces rapid loss of flight within minutes (25% within 5 min, 50% within 10 min, 100% in 25 min; blue line) and leads to 99% mortality within 18 h (red dashed line). The hornets were dusted 3 times with activated charcoal within 5 min and then again after 20 min (dashed vertical line), corresponding to the treatment of a nest. Note that the x-axis is logarithmic.

Discussion

This is anecdotal evidence only, as we only treated one nest and do not have a sham-treated control group. Any observation may be for other reasons than dusting with activated charcoal, e.g. stress of transfer to the flight cage. We report those observations nevertheless in the hope that this experiment will be replicated.

References

[1] I. Pérez-de-Heredia, E. Darrouzet, A. Goldarazena, P. Romón, and J. C. Iturrondobeitia, "Differentiating between gynes and workers in the invasive hornet *Vespa velutina* (Hymenoptera, Vespidae) in Europe," *J. Hymenopt. Res.*, vol. 60, pp. 119–133, Oct. 2017, doi: 10.3897/jhr.60.13505.
