# Supplementary figures and images for: Activated Charcoal: A Highly Potent Legal Alternative for Vespa velutina Nest Destruction
Source: Insects. 2026 Apr 9;17(4):407. doi: 10.3390/insects17040407 (PMC13115649; doi:10.3390/insects17040407)

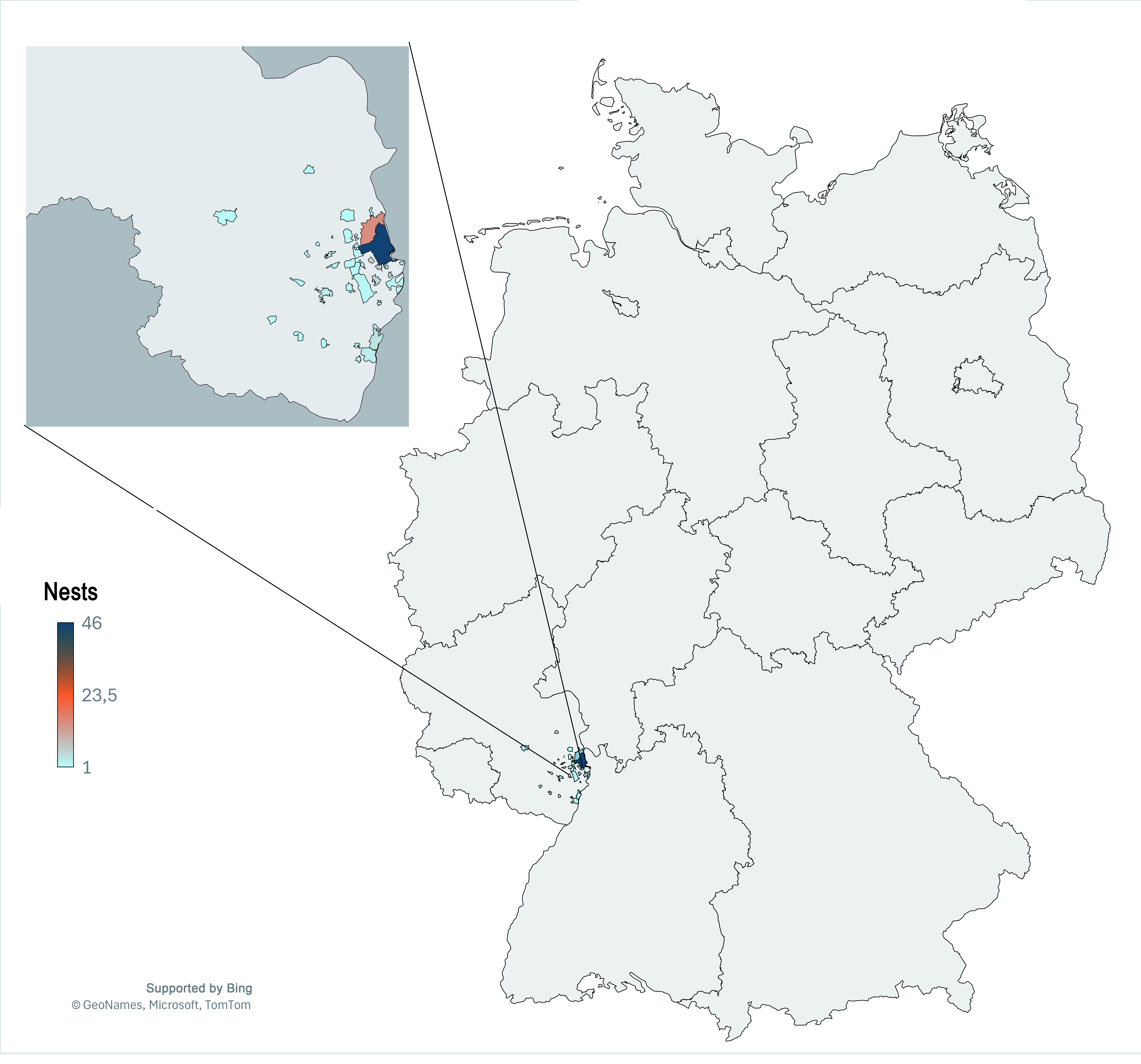

Supplement: Supplementary file 1 [file insects-17-00407-s001.zip › Supplemental_figure_S3.jpg]

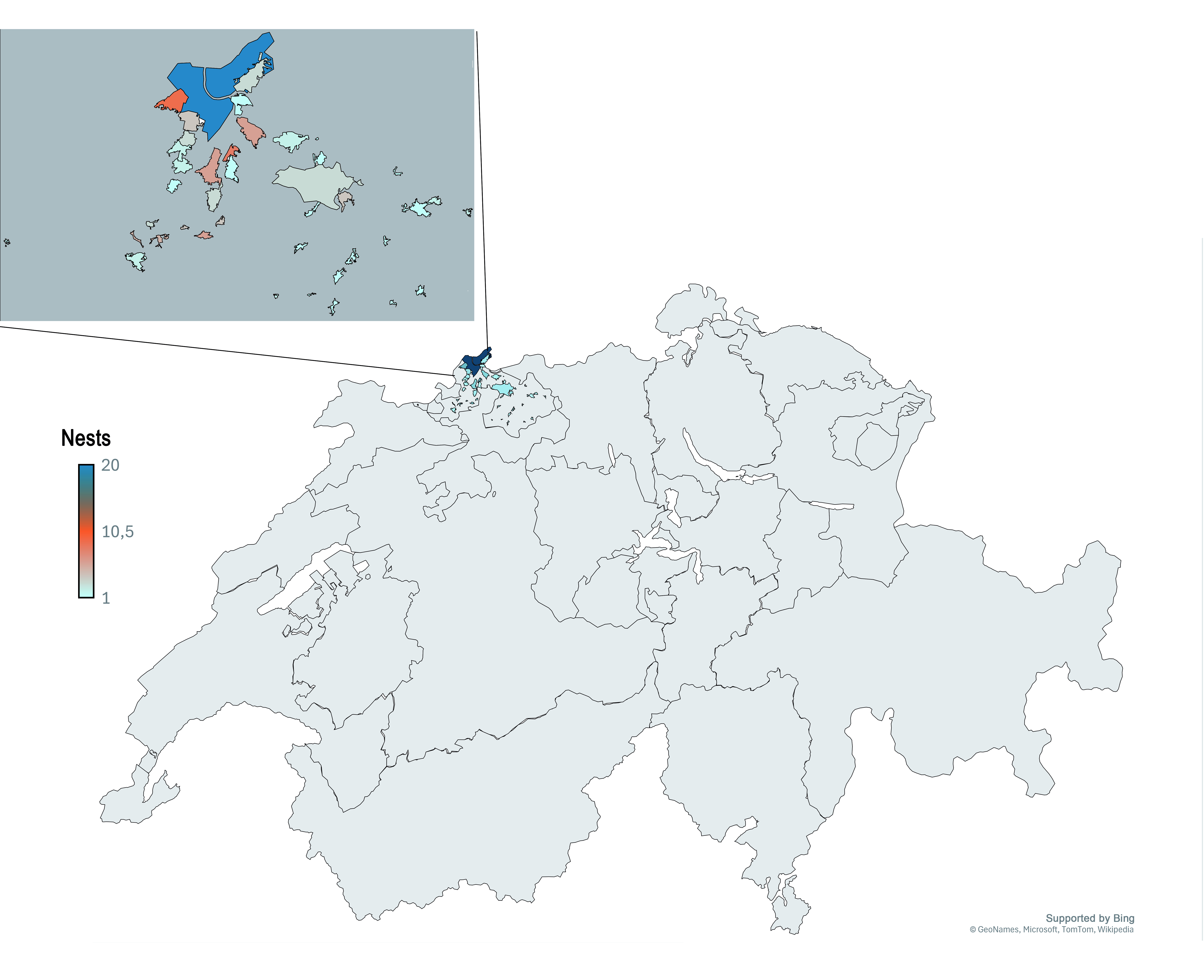

Supplement: Supplementary file 1 [file insects-17-00407-s001.zip › Supplemental_figure_S2.jpg]

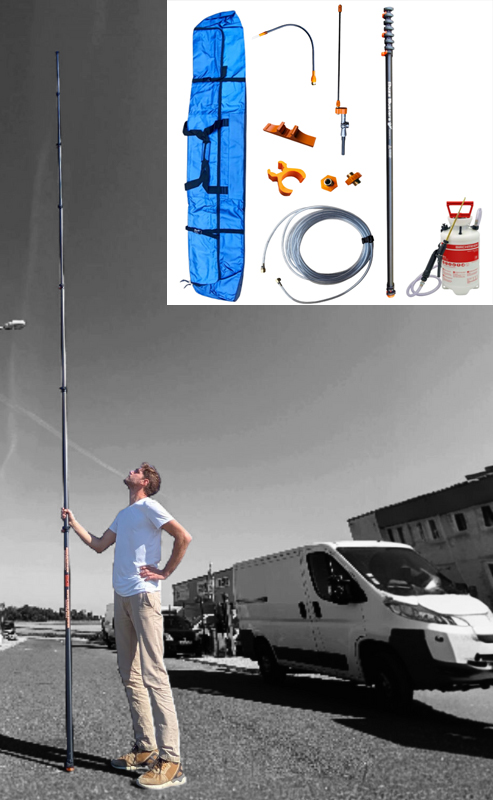

Supplement: Supplementary file 1 [file insects-17-00407-s001.zip › Supplemental_figure_S1.jpg]
